# Early observations of Tier-3 drug shortages on purchasing trends across Canada: A cross-sectional analysis of 3 case-example drugs

**DOI:** 10.1371/journal.pone.0293497

**Published:** 2023-12-21

**Authors:** Araniy Santhireswaran, Cherry Chu, Katherine Callaway Kim, Étienne Gaudette, Lisa Burry, Fiona Clement, Katie Suda, Mina Tadrous

**Affiliations:** 1 Leslie Dan Faculty of Pharmacy, University of Toronto, Toronto, Ontario; 2 Women’s College Hospital, Toronto, Ontario, Canada; 3 Center for Health Equity Research and Promotion, VA Pittsburgh Healthcare System and Division of General Internal Medicine, University of Pittsburgh Schools of Medicine and Pharmacy, Pittsburgh, PA, United States of America; 4 Institute of Health Policy, Management and Evaluation, University of Toronto, Toronto, Ontario, Canada; 5 Lunenfeld-Tanenbaum Research Institute, Mount Sinai Hospital, Sinai Health, Toronto, Ontario, Canada; 6 Department of Community Health Sciences, Cumming School of Medicine, University of Calgary, Calgary, Alberta, Canada; Sergio Arouca National School of Public Health: Escola Nacional de Saude Publica, BRAZIL

## Abstract

**Background:**

To curb the growing impact of drug shortages, Health Canada developed the Tiered Notification and Communication Framework which assigns potential shortages a corresponding tiered status. Tier-3 is assigned to shortages with the greatest potential impact on the healthcare system. This study aims to describe drug purchasing trends in response to Tier-3 shortages using three case-examples.

**Methods:**

We conducted a time-series analysis of monthly purchasing data for three out of 17 Tier-3 drug shortages (hydralazine, sarilumab, and medroxyprogesterone acetate) with publicly available reports in July 2021 and available IQVIA MIDAS data from January 2016 to December 2021. We assessed percent changes in purchasing at 1-, 3-, and 6-months after the onset of each Tier-3 drug shortage and interventional ARIMA modelling was used to assess the statistical significance.

**Results:**

Medroxyprogesterone acetate experienced a significant shift (p = 0.0370) in purchasing following its shortage, and the 1-, 3-, and 6-month percent changes were +14.9%, +6.8% and -3.1%, respectively. Hydralazine and sarilumab did not show a significant shift. The 1-, 3-, and 6-month percent changes for hydralazine were +15.5%, +10.2%, and +9.6% respectively and +25.2%, +45.1% and +39.2 for sarilumab.

**Conclusions:**

These results indicate that drugs assigned a Tier-3 status may not show declines in purchasing in the months following status assignment, which may be due to policy responses following the assignment. However, more insight is needed into the mechanisms through which these policy measures impact shortages and whether they are functioning as intended.

## Introduction

Drug shortages are a growing concern within health systems globally. In Canada, this continues to be an emergent and troubling issue [1]. Canadian manufacturers reported on average 238 new shortages per month between 2017 and 2020 [2]. Disruptions to drug availability negatively impact patient outcomes and strain the healthcare system [3]. For example, the recent valsartan recall was estimated to affect 160,000 Canadians within three months, causing challenges with continuing therapy, and in turn, negatively impacting clinical outcomes and further burdening the healthcare system [4].

The recent COVID-19 pandemic has exposed and amplified weaknesses within the drug supply chain [5,6]. Disruptions in the supply chain at the start of the pandemic raised concerns of increased frequency and extent of drug shortages [5]. To address rising drug shortages and concerns that supply disruptions would worsen during the pandemic, Health Canada, Canada’s drug regulator, developed a number of policies to curb the number and impact of shortages. One of the major policy enactments was the development of the Tier Assignment Committee (TAC) in 2017, which consists of federal and provincial governments and healthcare representatives [7]. TAC focuses on assessing the potential scale and impact of a shortage and assigning a corresponding tier status using the Tiered Notification and Communication Framework [7]. Tier-1 emphasizes the earliest notification of a shortage and is assigned to all anticipated drug shortages. Tier-2 captures all actual drug shortages where therapeutic alternatives are still available. Tier-3 status is assigned to shortages without an available therapeutic alternative, which leave the greatest potential impact on the healthcare system [7,8]. Drugs at risk of Tier-3 shortages are eligible for special policy measures such as exceptional importation and sale of a foreign-authorized drug [9]. Unique Tier-3 policy measures are intended to maintain drug supply and access for these potentially severe drug shortages.

The impact of Tier Notification and Communication Framework policies on post-shortage drug utilization is not well understood. Many similar policies and frameworks have been implemented globally to help mitigate the impacts of shortages [10–12], and understanding the impact of these policies is needed to help inform future work to continue to battle the ongoing supply chain problems. In this study we described changes in purchasing trends for some early cases of Tier-3 drug shortages, using monthly purchasing data from IQVIA MIDAS (all rights reserved) as a proxy. We conducted observational case-studies of three different Tier-3 drug shortages to better understand the impacts of Tier-3 policies and whether they can mitigate severe drops in drug access as intended.

## Methods

### Data source

We leveraged the IQVIA Multinational Integrated Data Analysis (MIDAS) database for country-level drug purchasing data for Canada. The IQVIA MIDAS dataset reports monthly purchases from health-system and retail pharmacies and major retail outlets (mass merchandisers, grocery stores, and convenience stores without pharmacies) for prescription and over-the-counter drugs by country [13]. Drug purchases are reported in aggregate for each country in terms of standardized units (SU), where a single SU represents the smallest common purchased unit for the given product (e.g., one oral solid tablet, capsule, one IV vial, or 5mL oral liquid) [14].

### Case examples- drug selection

All 17 Tier-3 drug shortages reported by Health Canada that were posted on the drugshortages.ca website during July 2021 were searched by drug name in the molecule and product search of IQVIA MIDAS. Drug shortages that occurred less than 6 months before the end of our data (December 2021) were omitted from analysis (n = 14). Only three drugs had sufficient post-shortage data to analyze the effects of shortage on drug purchases: hydralazine, sarilumab, and medroxyprogesterone acetate. The hydralazine solution shortage occurred on August 21st, 2019 due to an increase in drug demand. The sarilumab powder shortage was reported on March 3rd, 2020 as a result of production delays and was resolved two days after. The medroxyprogesterone acetate 150 mg suspension, which is the only strength available in Canada, faced a shortage on July 6th, 2021 in response to disruptions in drug manufacturing [15]. These three drugs were selected as case examples given data availability and sufficient post-shortage monthly data to accurately examine the shortage impact.

### Study design & statistical analysis

To assess the changes in purchases before and after the shortages, a time-series analysis of monthly purchasing data for each drug was conducted from January 2016 to December 2021. Changes in drug purchases were quantified as changes in total standardized units (SU) at 1-, 3-, and 6-months post-shortage compared to the same time-period from the year prior.

Interventional-Autoregressive Integrated Moving Average (ARIMA) modelling was used to assess the statistical significance of Tier-3 shortage onset on drug purchasing trends [16]. ARIMA models are a type of time series analysis which can be used to study whether interventions or events were associated with significant changes in longitudinal data. ARIMA modeling accounts for seasonality and autocorrelation, making it ideal for studying drug purchasing trends [16,17]. We hypothesized that purchases would gradually decrease at the start of the shortage due to a lack of drug availability. Therefore, we fit a “ramp” intervention to evaluate long-term changes in drug purchases starting from the drug shortage start date [18]. Sarilumab and medroxyprogesterone acetate were first-differenced to establish stationarity and stabilize variability due to yearly patterns [15].

To optimize model fit, we added moving average (q) and autoregressive (p) terms as appropriate based on residual autocorrelation function, partial autocorrelation function, and white noise probability plots. Statistical analysis and ARIMA modelling were conducted using SAS version 9.4.

## Results

**Fig 1:**
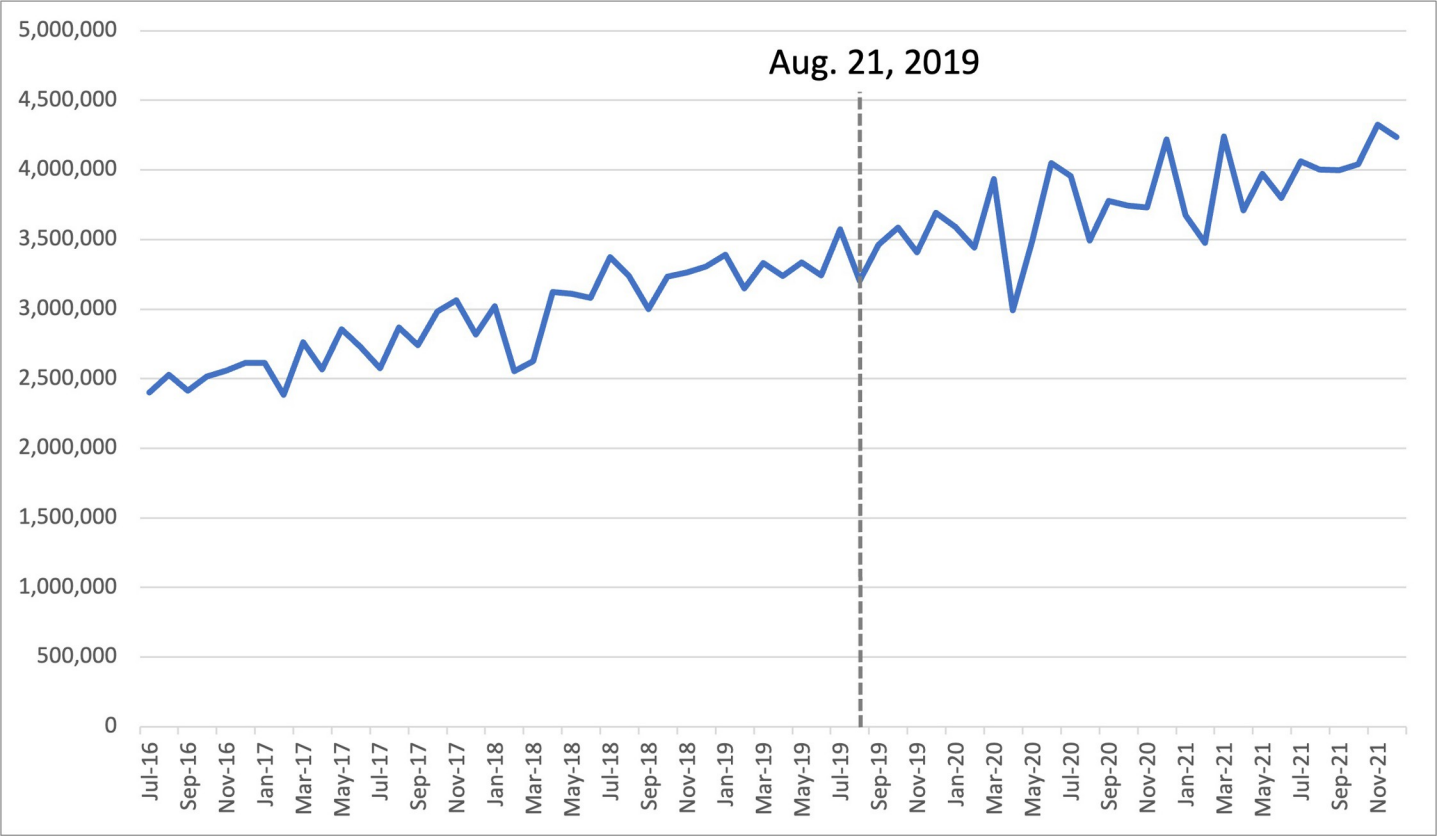
Trends in monthly purchases for hydralazine between Jan 2016 –Dec 2021 in Canada. Hydralazine did not experience a significant shift in drug purchasing trends, but the percent changes and figure indicate a steady increase. The range of drug purchases for hydralazine prior to the shortage was 2,401,820 SU to 3,574,760 SU, and 2,991,680 SU to 4,323,620 SU after the shortage.

The range of drug purchases for hydralazine prior to the shortage was 2,401,820 SU to 3,574,760 SU, and 2,991,680 SU to 4,323,620 SU after the shortage, with a pre-shortage average of 2,923,649 SU and 3,769,401 SU post-shortage. The 1-, 3- and 6-month percent changes for the post-shortage periods compared to the year prior were 15.5% increase (+463,940 SU), 10.2% increase (+965,960 SU) and 9.6% increase (+1,566,510 SU), respectively. This indicates an increase in purchases compared to the same time periods from the year prior to the shortage (Table 1). Hydralazine purchasing trends did not experience a significant shift in response to the shortage as indicated by ARIMA analysis (p = 0.8933) (Fig 1).

**Table 1:** ARIMA values and percent changes in purchasing trends for Tier-3 drug shortages.

Drug Name	Shortage Date	Reason for Shortage	ARIMA p-value (ramp)	1-month	3-month	6-month
Hydralazine	Aug. 21^st^, 2019	Demand increase	0.8933	15.5%	10.2%	9.6%
Sarilumab	Mar. 3^rd^, 2020	Production delay	0.2454	25.2%	45.1%	39.2%
Medroxyprogesterone Acetate	Jul. 6^th^, 2021	Manufacturing issues	0.0370	14.8%	6.8%	-3.1%

**Fig 2:**
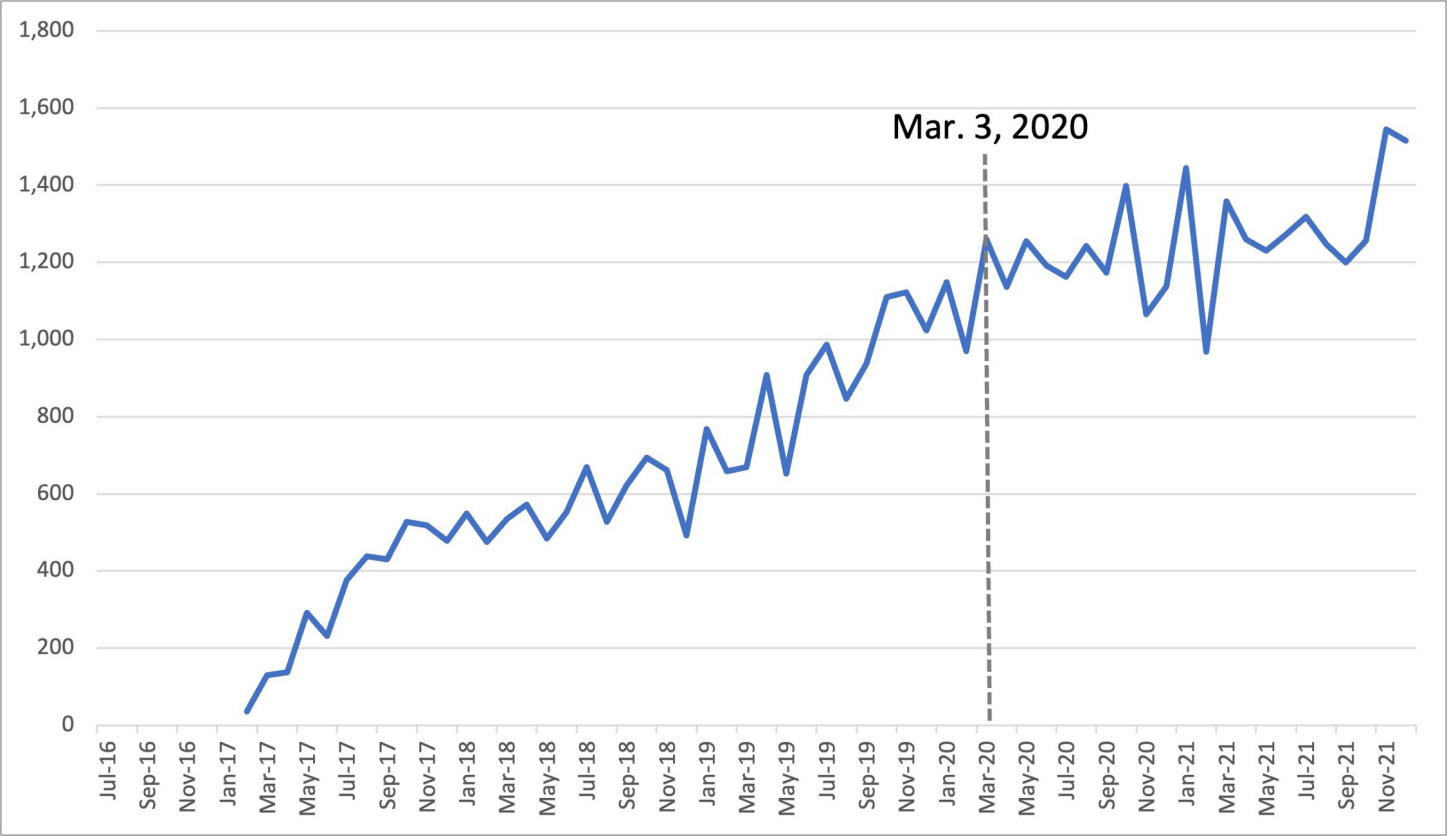
Trends in monthly purchases for sarilumab between Mar 2017 –Dec 2021 in Canada. Sarilumab purchases did not experience a significant shift as indicated by ARIMA analysis. The percent changes indicated an increase in drug purchases, where the range of drug purchases was 36 SU to 1,149 SU, and 968 SU to 1,545 SU following the shortage.

For sarilumab, prior to the shortage the range of drug purchases was 36 SU to 1,149 SU, and 968 SU to 1,545 SU following the shortage, with a pre- and post-shortage average of 626 SU and 1,256 SU, respectively. The 1-, 3- and 6-month percent changes for sarilumab were 25.2% increase (+229 SU), 45.1% increase (+1,114 SU) and 39.2% increase (+1,685 SU), respectively, indicating an increase in purchases compared to the year prior (Table 1). ARIMA modelling indicated no significant changes in purchasing trends (p = 0.2454) (Fig 2).

**Fig 3:**
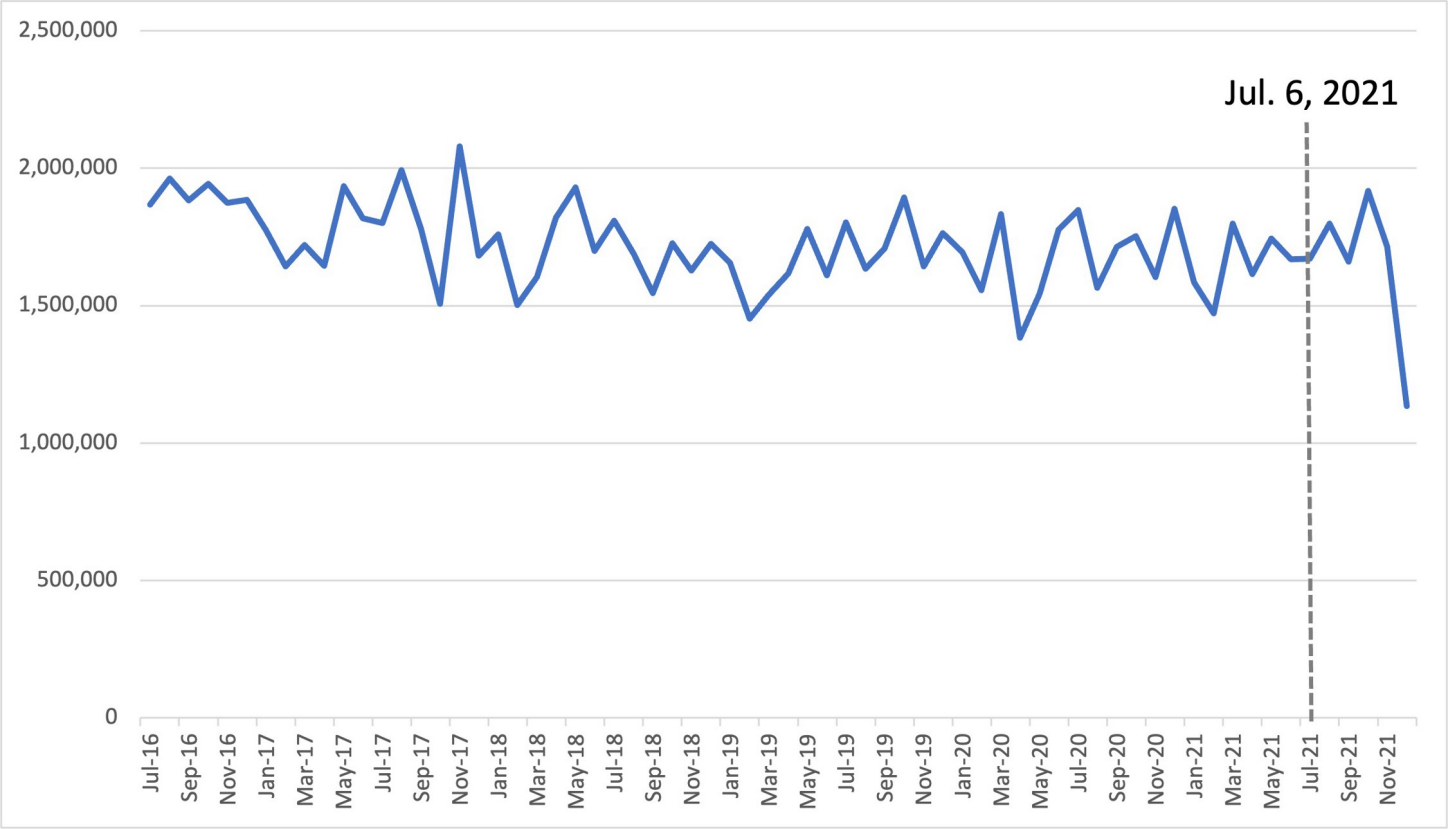
Trends in monthly purchases for medroxyprogesterone acetate between Jan 2016 –Dec 2021 in Canada. Medroxyprogesterone acetate did encounter a significant shift in drug purchasing trends as illustrated by ARIMA analysis. The range of drug purchases prior to the shortage was 1,383,928 SU to 2,080,376 SU, and 1,134,681 SU to 1,917,025 SU after the shortage. The 6-month percent change indicated a decrease in drug purchases by 3.1% compared to the year prior.

The range of drug purchases for medroxyprogesterone prior to the shortage was 1,383,928 SU to 2,080,376 SU, and 1,134,681 SU to 1,917,025 SU after the shortage, with an average of 1,722,218 SU before and 1,649,363 SU after. The percent changes for 1-, 3-, and 6- month periods post-shortage were 14.9% increase (+232,697 SU), 6.8% increase (+341,034 SU) and 3.1% decrease (-265,032 SU), respectively, indicating an increase followed by a decline in drug purchases relative to the year before (Table 1). Interestingly, the decrease in purchases only occurred in the 6^th^ month after the shortage onset (Fig 3). ARIMA analysis confirmed a significant downward shift in 6-month purchases relative to historical trends (p = 0.0370).

## Discussion

Of the three Tier-3 shortages case-examples observed only that of medroxyprogesterone exhibited a significant decline in purchases. The other two, for hydralazine and sarilumab, were associated with a continued increase in purchases in line with their historical trends. More importantly, hydralazine and sarilumab did not encounter a severe or persistent reduction. These results suggest that although drugs assigned Tier-3 status may face shortages, policies in place may be effective in curbing severe drops in drug availability. Given that Tier-3 drug shortages are at risk for high impact on the healthcare system, the absence of severe reductions in drug purchases is reassuring.

Currently, there is limited research on drug utilization during shortages and the effects of drug shortage policy measures [19,20]. Previous studies have investigated the impact of specific global shortages on utilization trends of related drugs. A recent study assessed the impact of the 2018 global valsartan recall and consequent shortage on Canadian hypertensive drug utilization trends. Findings illustrated substantial reductions in valsartan use and cascading effects on other antihypertensives, although the mechanisms behind this global shortage were not understood [21]. Global valsartan purchases decreased by 15.7% (difference = -61 166 515 SU), and global non-valsartan angiotensin receptor blocker purchases increased by 44.8% (difference = +957 069 420 SU) [21]. A Canadian federal government report quantified changes during shortages between fiscal year 2017 and 2019, prior to the COVID-19 pandemic. It found that 8% of shortages were followed by a steep decline in beneficiaries accessing their drugs when considering substitutions to similar drugs [2]. Studies have also assessed the impact of specific policy measures on drug shortage prevalence, where results indicated only temporary reductions in shortage rates [20]. Many studies have also assessed the impact and performance of many global policy measures, such as, the National Surveillance Center and therapeutic interchange policies in South Africa [21,22]. Collectively, these findings highlight the need for consistent monitoring of drug shortage rates and consequent utilization trends, along with ongoing assessment of the effects of implemented policy measures [20]. Future studies should also focus on differing drug shortage policies and quasi-experimental methods, for a more holistic understanding of shortage mechanisms and the causal impact of specific policies. Insights into the effects of these policies will help improve the selection of appropriate policy levers for differing types of drugs and shortages.

The development of tiered drug lists is an important and novel approach to helping curb the impact of drug shortages. In addition to coordinating communications across key stakeholders, Tier-3 drugs are eligible for unique interventions to safeguard drug supply. One regulatory intervention is the exceptional importation and sale of foreign-authorized drugs that are otherwise not licensed for sale in Canada [5,8]. Tier-3 drugs are also eligible for expedited regulatory reviews allowing for accelerated market availability [8,9]. Moreover, to increase drug access, the shelf life of a drug can also be extended if there is supporting data [5,8]. However, the explicit interventions used for specific drug shortages are not publicly shared, making it difficult to study the distinct impacts of each intervention. Although the primary intervention implements during Tier-3 shortages is mandating manufacturers to maintain a buffer stock of the drug prior to the shortage. This allows for uninterrupted supply throughout the shortage. These interventions are aimed at reducing the impact of Tier-3 shortages on drug availability, and possibly contribute to explaining the minimal reductions in drug purchases seen in the three drugs studied. However, there are risks with these interventions, such increased drug spending due to switching to higher cost alternatives which may be exasperated by the lack of pricing agreements and negotiations. These unique interventions are crucial given that Tier-3 drugs lack available therapeutic alternatives, and clinicians feel that other treatments are suboptimal.

Similar interventions have also been implemented in countries across the world [10–12]. For example, almost all European countries have a publicly available database for drug shortage reporting, and organizations aimed at gathering information on shortages. Many European and non-European countries have also implemented bottom-up initiatives including new shortage guidelines, codes of conduct, taskforces, management plans, manufacturer surveillance and improved reporting [10,11]. Given the novelty and intricate nature of these policies, it is crucial to understand their impact and function in mitigating shortages. Moreover, it is crucial to consider the country-specific clinical importance and application when studying the shortage impacts of drugs across the globe. Our work helps offer insights into the impact of policy measures and shortages on specific drugs, however, it is difficult to investigate the effects on all drugs in this manner. Therefore, it is crucial to compile a list of important and at-risk drugs and study the impact of drug shortages and policy measures on their use and access. Future work should also aim to compare these at-risk drugs to those not on the list to better understand predictors of shortage risk. Overall, compiling an at-risk drug list and understanding important shortage predictors can provide the necessary real-world evidence to further inform policymaking.

We acknowledge limitations to this study. The methods used in this study were observational in nature and cannot establish a causal link between policies associated with Tier-3 assignments and outcomes. Because only 3 of the 17 drugs with reported Tier-3 shortages in July 2021 had enough post-shortage data to be included in the analysis, the number of case studies conducted was small and findings may not be generalizable to other shortages [7]. The utilization trends of more Tier-3 shortages need to be studied to gain a comprehensive understanding of Tier-3 policy effects on drug use and shortage outcomes. Additionally, future work must study if the policy helped reduce the extent of shortages compared to prior periods of time or compared to drugs that did not have tier 3 status. In addition, patient-level fill data was unavailable, which prevented us from examining sociodemographic characteristics and clinical information associated with drug purchasing trends. The lack of information on patient diagnoses and outcomes prevented us from understanding whether the reasons for drug discontinuation were clinically appropriate, due to adverse events, because of the shortage, or unrelated to the shortage. Additionally, the data only represents the volume of drugs purchased, but we could not confirm whether the drug was consumed or not. As a result, the number of patients affected is unknown.

## Conclusion

Drug shortages represent a growing concern that strains healthcare systems and harms patient care globally [6]. Our results offer early observations that policies used in Canada in conjunction with Tier-3 assignments may potentially help curb severe drops in drug utilization, but little is understood about the actual mechanisms behind drug shortages and the impact of specific policy measures. Through more targeted research and policy improvements, it may be possible to minimize the harms of drugs shortages for both patient care and professional practice [20].
